# Observations on the Effects of Opium on the Human Body

**Published:** 1803-11-01

**Authors:** F. Weber

**Affiliations:** Foreign Depot, at Lymington, Hants


### Observations on the Effects of Opium on the Human Body, *by* Mr. F. Weber, *of the Foreign Depot, at Lymington, Hants.*

TO acquire a perfect knowledge of the effects of opium on the human body, I have taken it myself for a length of time in all quantities, as far as prudence would admit, and under different circumstances; I have administered it to two medical friends, and to a number of persons in health in the same manner, and have observed its effects with attention on patients labouring under local and general diseases, to whom it had been administered as a medicine for various purposes.

From any single dose, sufficiently strong to produce sensible effects, I always experienced first certain sensations in the forehead, a kind of pressure and straining, the latter of which seemed to reach down to the eyes and nose, attended by an increase of vigour, confidence, and cheerfulness. Here it would remain had the dose been within two grains of solid purified opium. The effects would last an hour or two, perhaps with some dryness in the mouth and fauces, and then vanish, leaving sometimes a slight degree of costiveness behind.

If I had taken two or three grains, the said sensations in the forehead would insensibly increase to a slight confusion of thought and giddiness, a heat and sensible pulsation of the arteries in the head; flushing of the face; a sensation of the eyes, as if they were swelled and the sockets not large enough to contain them; a slight degree of sickness; a dryness of the mouth and fauces; slightly diminished sensibility, and propensity to sleep; sometimes gripings and a strong contraction of the intestines.

These effects lasted an hour or two, whilst an elevation of spirits and watchfulness remained yet for an uncertain time behind; sometimes a few hours, at other times the whole remaining part of the day; as did likewise costiveness.

From four to six grains caused all the named effects in the same order, but in a higher degree and quicker succession. The pressure in the forehead increased often quickly and passed on to pain; there was also an itching in the nose, and not very rarely all over the body; hiccup, stupor, and strong propensity to sleep; startings, and a strange chaos of happy and disagreeable visions, if sleep sometimes surprised me whilst I was sitting in a chair, and a strong increase of confusion and giddiness, and sometimes when I recollected myself again, sickness and vomiting; impaired voluntary motion; stammering in speech and trembling; heavy uneasy eyes; contracted pupils and dimness of sight; finally, a dysuria with frequent involuntary interruptions in the stream of urine, costiveness cum gradu impotentiae. It is a curious, but not the less certain fact, that through all the stages of the effects of opium, the most cheerful flow of spirits not excepted, I perceived the latter phenomenon, which undoubtedly was owing to a want of due sensibility. I experienced these effects from four hours to a whole day, and sometimes longer, and a liveliness, activity, and watchfulness, rather in a higher degree than usual, which succeeded the former affections, viz. giddiness, stupor, sickness, &c. and seemed to be proportionate to them.

A lassitude and exhaustion, and strong propensity to sleep, were the late and last effects which I experienced, more or less, sooner or later, in proportion as the earlier effects had been more or less strong, and lasted a shorter or longer time. If, for instance, I had taken from four to six grains, at ten or eleven in the morning, I sometimes experienced a burning heat creeping throughout my limbs, but which was particularly strong in the palms of the hands, followed by exhaustion, at nine, ten, or twelve, or there abouts, at night; sometimes I experienced lassitude and propensity to sleep next morning, when I got up, at eight or nine; sometimes at noon, or any other time during the the day or at night. I cannot exactly determine how long this state of relaxation lasted, but I think I sometimes felt it on the third and fourth day, after having continued most part of the second, or at least made its appearance on that day. From seven to ten grains caused, besides the former effects in a proportionate high degree, a distressing sickness and vomiting; violent gripings, lassitude, stupor and sopor, which continued a whole day.

Sometimes, if I had taken seven or eight grains only, I would be quite well the next day, if a sickness and even vomiting directly after breakfast did not remind me, that some effects were yet remaining. If I had taken full ten grains at once, I perceived the first day, giddiness, trembling, stupor, dimness of sight, great difficulties in making water, violent gripings and vomiting, if I ate or drank any thing; afterwards, continual but highly disturbed dosing.

Next day, though much milder, I perceived most of the said effects. On the third day I was well, active, and cheerful. On the fourth day I began to be despondent, tired and exhausted, and continued in that state to the sixth and even seventh day.

All the different doses of which I have enumerated the effects, I took, from one to three hours after breakfast, that is, between nine and twelve o'clock.

Not to use myself too much to opium, I sometimes left it off for a week; besides that, if I had taken a strong dose, I waited four, five, or six days without taking any, in order that no effect should escape me.

Half a grain of solid opium, taken every half hour; or a grain taken every hour; or a grain and a half taken every hour and a half, or every two hours, has insensibly brought on a happy humour, increased vigour, activity, and unusual watchfulness, which I perceived after having taken about two, three or four grains, and which continued if from time to time I repeated the same procedure for several days and nights, without the least inclination to sleep, without tiredness, or any impaired function, except dryness in the mouth and fauces, and thirst, occasionally gripings, and costiveness. I have made this experiment often, being a very pleasant one, and only the consideration of injuring my health, prevents me from repeating and persevering in it longer. When I discontinued the use of opium, taken in this manner, I perceived the second or third day after, the same stupor and exhaustion as I had experienced when I had taken the same quantities, which I had now used in small doses, at one, two, or three different times.

The longer the use of opium had been continued, and the greater the quantities which had been taken, the greater was the ensuing exhaustion, as might a priori be supposed, and the longer it lasted. If I had taken opium for a week or longer, from six to ten grains per day, and then suddenly discontinued it, the exhaustion and stupor would, though gradually diminishing, continue for three or four days, during which time I was unfit for any business. Once, when I had taken from eight to ten grains every day for about a fortnight, I perceived the second day after I had left it off, besides a high degree of lassitude, violent pains in the knee and hip joints, which became so excruciating at night that I could neither sit nor lie.

Determined at first to wait the event, I supported the pains till they grew so violent that my fortitude failed me, and I had again recourse to a dose of opium. At twelve o'clock at night a medical gentleman, who was with me, gave me eight grains in two pills, and in three quarters of an hour I perceived the usual sensation in the forehead, a glow in the face and head; my spirits began to raise; sensibility sunk, and ere I was aware of it all my pains had vanished. I blessed the power which I had cursed the moment before; pleasant fancies floated in my mind, and deprived me of sleep.

If at any time, in any degree of the said exhaustion and despondency, I took four, six, or eight grains at once, or in repeated doses; vigour and activity, and a disposition of the mind to happy reasoning took place, as soon as the opium began to operate.

I have taken opium for three weeks and never experienced any symptom of lassitude and exhaustion, (as the late conequence of former effects) because I at first took the same quantity every day, and afterwards increased the dose to two or three grains in the whole. I used to get up perfectly well and in good spirits, and remained so all the time till I took another dose, which was generally at ten, eleven, or twelve o'clock. The effects of solid opium, taken in pills, at the mentioned time, I could never perceive, before three quarters of an hour, sometimes a full hour, and not very rarely an hour and a half had elapsed.

Nor have I ever been able to discover any alteration in the pulse or general temperature of the body (though I have paid the strictest attention to both), which I could have attributed to a direct operation of the opium on the heart heart and blood vessels. Neither have I found that opium affected the processes of the economy at large, nor, as will be perceived, was the appetite particularly impaired, those intervals excepted where actually sickness and vomiting, or high degree of stupor, or sopor, or exhaustion had been produced.

It will be superfluous to mention, that, in as far as opium causes rest and sleep, it must materially reduce the frequency of the pulse, the circulation being more slow during the latter than when the body is in a watchful and active state. The contrary takes place during liveliness and activity.

This is to be applied also to the general temperature of the body, and processes of the economy. Dissolved in any usual menstruum, opium produced effects in half an hour.

If taken with a small quantity of spirits or any liquid containing alcohol, or any warm fluid, the effects made their appearance sooner than usual.

Any meal taken withing half an hour or later after a dose of opium, speedily brought on all its effects, and it seemed in a considerable higher degree. Gripings, sickness and vomiting, I once or twice experienced directly after breakfast, having taken a strong dose the evening before. If taken within an hour after dinner, the effects appeared later than usual and were less in degree; I even sometimes could hardly perceive them at all, if the dose had been a moderate one.

The effects seemed to be the stronger, the more early in the day the opium had been taken. They were strongest if I were unwell, or if any thing had in the least deranged the functions of the economy; for instance, if I had been up all night, if I had been sick and vomited, and taken no food, two or three grains would have as much effect as otherwise four or five.

The effects were the stronger, the shorter the time since a former dose had been taken, even if the effects caused by the latter seemed to have subsided; if, for instance, I had taken two or three grains at ten or eleven o'clock in the morning, and giddiness, confusion, &c. seemed to have ceased at three, four, or five in the afternoon, a repeated dose would yet produce much stronger effects than the first. Again, if a grain or a grain and a half had produced little or no effect, the same quantity administered an hour or two after, caused exhilaration, pressure in the forehead, dryness in the mouth and fauces, &c.

 If

If I was in high spirits and very active; if I walked, or took such exercise as made me warm and perspire, the effects made their appearance considerably sooner, were less in degree and much less in duration. I have walked to Southampton, a distance of four miles from my former residence at Totton, (a small village) in a hot day, and whilst I was very warm and strongly perspiring, I took successively three pills on the road, each pill of 4 grains; so, that having taken a similar one half an hour before I set out, I took sixteen grains in about an hour and an half, of which I perceived much less effect, than when I took four or six grains at my usual hour coolly and quietly at home. Having been about an hour in Southampton, I walked home again, and I must confess that I did not feel the least effect remain whatsoever, not even elevation of spirits and watchfulness, but on the contrary I was tired and exhausted, as I should have been at any other time after such an excursion. The reader will perceive that this was at a time when I was in some degree used to opium. But I have made, I may say, a hundred similar trials which confirm the above. I have frequently taken the same quantity at the same hour as I did when I remained at home, and directly after walked out, or set off at a brisk pace on horseback, and always perceived that the effects made their appearance much sooner, were much less in degree, and lasted, instead of eight or ten, only two or four hours.

Under the influence of emotions, strong sensations, and any particular strong attention and exertion of the mind, the effects of opium appear later than usual, are less in degree, and much shorter in duration.

If it so happened, that the cause which disturbed my mind was removed, then all the effects would almoft instantly make their appearance and take their usual course.

Cold, rest, but particularly sleep, also retard the appearances of the effects of opium, and prolong them.

If I had taken any quantity during the day, in consequence of which only slight effects remained when I went to bed; in that case sleep would be the *means* of restoring perfect order to the functions of the economy.

Four grains of jalap taken with an equal quantity of opium in four pills, has caused several stools and the usual effects of the latter.

I have often repeated this, and generally perceived more purging than I should have supposed from so small a quantity of jalap, and in such a combination.

The evacuation of the stomach by irritating the orifice of of the pharynx with a finger, the moment when the effects made their appearance, or before, has cut short the effects of any dose. Beyond an hour after the effects had made their appearance, I have found vomiting excited in the same manner, not alone useless, even if the stomach was quite emptied, so that nothing but bile was brought up, but even aggravating confusion, giddiness, stupor, and particularly retching to a high degree.

Vegetable acids, especially lemon juice, if used in sufficient quantity and for a sufficient length of time, before the opium has been carried into the circulation and produced strong effects on the head, resist its operations in a great measure, but do not quite counteract them. I have taken the juice of a lemon diluted with half a tumbler of water, at the same time with eight grains of opium; I have repeated the draught five times in three or four hours, then discontinued it, and in an hour after I have experienced all the usual effects of opium. I have taken at another time the same dose of opium with the same quantity of lemon juice, repeated the latter at first every half, and afterwards every three quarters of an hour, for five or six hours, and the effects of the opium was inconsiderable in proportion to what it used to be.

About two ounces of vinegar used as the former, has done me much the same service. Vegetable acids, like vomiting, are useless and hurtful, if confusion, giddiness, sickness, &c. have lasted some time. Immediate vomiting and violent retchings are the infallible consequences without affording any relief. Mineral acids possess no property at all in counteracting or removing the effects of opium,

A tumbler full of warm milk, drank directly after it came from the cow, took the gripings away instantaneously, as often as I used it for that purpose.

To a medical friend of mine, who wished to take a dose of opium for the sake of observation, I gave three pills, containing together three and a half grains of opium and three grains of jalap. An hour after (about seven o'clock in the evening) he got sick and giddy, having drank tea shortly before.

In about another hour the said effects had increased to such a degree, that he was obliged to go to bed. He was not much disturbed in the night, and got up tolerably well next morning. He made a hearty breakfast, consisting of coffee and toast; but got giddy, sick, and vomited soon after. He was again obliged to lie down, and remained in bed the whole day. I administered to him, from time to time, the juice of a lemon diluted with half a tumbler full full of cold water; but he soon perceived, that far from relieving him, this caused an increase of sickness and vomiting, and violent retchings. As often as he made water, he experienced the abovementioned difficulties, and he experienced also some griping.

Directly after he had taken tea in the evening, not having been able to make any dinner, he vomited, and perceived renewed gripings. He got up seemingly well next morning, after having enjoyed a good night's rest, but found himself unwell, giddy, and rather sick for some time after breakfast; which, as the day before, consisted of coffee and toast. He perfectly recovered during this, and continued in good spirits till the fifth day, when he began to feel himself very tired and despondent.

On the following day he was so sleepy, that every effort to read was in vain; and it was not till some days after that he enjoyed his usual alertness again.

Another medical gentleman took early in the morning, before breakfast, a pill of one grain. Not perceiving any effect, he took another grain an hour and a quarter after. Having waited again an hour and a half, without feeling the least effect, he took two pills of a grain each, which caused in less than an hour some giddiness; directly after head-ach, red and swelled eyes, strong inclination to sleep, and dryness in the mouth and fauces. After some time, he perceived violent gripings, which became easy directly after he had drank a tumbler full of warm milk as it came from the cow. About an hour after the gripings returned, and warm milk, used as before, afforded him the same relief. Five hours from the time that he had taken the last dose, he drank two dishes of tea, to which he ate some toast, which soon caused violent giddiness, sickness, and vomiting. Having lain down for an hour, he walked out, got warm, perspired, and came home perfectly well. He was costive this and the next day. After some time, he took again two grains very early in the morning, which produced no effect. Two hours after he repeated the same dose; from which he perceived nearly the same effects as before; only that he was near a quarter of an hour before he could make water, which was about five or six hours after he took the last dose.

Both gentlemen are between five and twenty and thirty years of age, of a common habit; that is to say, neither plethoric and very strong, nor weak and irritable. As for myself, I am of about the same age, and rather of that disposition which is pretty generally known in this country by the expression nervous.

A young

A young lady, lively, healthy, and strong, who was anxious to know what effect those pills could have, which I constantly carried about me, and which I took at all times, she could not conceive for what purpose, desired me to let her take some. Afraid of causing disagreeable and violent effects, I only gave her now and then, and at different times, and under different circumstances, one grain; of which, neither she, who waited with female curiosity, nor myself, could perceive any effect. One morning, in compliance with her ardent entreaties, I gave her two grains at breakfast, at seven o'clock. I did not see her again till evening, when she told me that she had been very sick *indeed*; very giddy and drowsy the whole day; but thought she was then quite well.

To eight men, in a general state of health, between five and twenty and fifty years of age, most of them attendants and the others patients in the Foreign Hospital, with local disorders, perfectly free from pain and fever, I administered to each four grains of opium, dissolved in a sufficient quantity of brandy, diluted with water, shortly before dinner.

In two hours after, two complained of gripings, giddiness, head-ach, drowsiness, thirst, and sickness; which lasted the whole remaining part of the day.

A third experienced gripings, and dryness in the mouth and fauces. Three others were slightly confused, giddy, and dry for some hours, and the remaining two perceived no effect at all.

Some of the former had more or less disturbed sleep, difficulties in making water, and costiveness. The others were well in the evening, and had a good night.

Sometime after I administered to three men, of the beforementioned age and healthy economy, a pill of three grains each, three hours and a half after dinner. In little less than an hour, two experienced a slight confusion of thought, slight giddiness, and grumbling motions in the bowels. In about half and hour more, the one perceived gripings, which, together with thirst and disturbed sleep, continued the whole night. I ordered him a quart of milk, several hours after the gripings had made their appearance, which he drank gradually without any benefit. The other had no gripings, but a grumbling in the bowels, giddiness, head-ach, thirst, costiveness, and slight difficulties in making water, which he experienced great part of the night, and a part of the next day.

The third experienced, very late, slight giddiness and thirst, and his sleep was rather disturbed.

[To be continued.]

